# Editorial: Bioinformatics and the Translation of Data-Driven Discoveries

**DOI:** 10.3389/fgene.2022.902940

**Published:** 2022-05-10

**Authors:** Asif M. Khan, Shoba Ranganathan, Prashanth Suravajhala

**Affiliations:** ^1^ School of Data Sciences, Perdana University, Kuala Lumpur, Malaysia; ^2^ Beykoz Institute of Life Sciences and Biotechnology, Bezmialem Vakif University, Istanbul, Turkey; ^3^ APBioNET.org, Singapore, Singapore; ^4^ Applied BioSciences, Macquarie University, Sydney, NSW, Australia; ^5^ Bioclues.org, Hyderabad, India; ^6^ Amrita School of Biotechnology, Amrita Vishwa Vidyapeetham University, Kollam, India

**Keywords:** bioinformatics & computational biology, knowledge discovery (data mining), biological data analysis biological databases data integration genome informatics genotype-phenotype relationships integrative data analysis machine learning multi-omics network analysis omics statistical methods systems biology, big data and artificial intelligence era, machine learning

Recent technological developments have given rise to multiple high-throughput biological data types, such as omics and other micro and macro-scale activities data, including those empowered by imaging technologies. Bioinformatics and computational biology approaches are key for analyses of large-scale datasets, invaluable for basic biological research and the translation of data-driven discoveries. The past 30 years have exemplified the evolving convergence of digital information, biological information, electronic medical records, and clinical information. The abundance of data and its exponential growth is a tsunami of opportunity for knowledge discoveries. For example, the European Bioinformatics Institute (EMBL-EBI), which maintains a comprehensive range of publicly available biological data resources, stored over 390 petabytes (10^15^) of raw data by the end of 2020 (Cantelli et al., 2022). In the next 5 years or so, we expect biological data to hit the exascale (10^18^). Big data, exhibiting the complex characteristics of 10 Vs (Suwinski et al., 2019), will require integration, inter-operability standardisation and implementation, the provenance of collected data, open data sources, open access to software, open-source software, machine learning and artificial intelligence, and massively parallel supercomputing.

**FIGURE 1:**
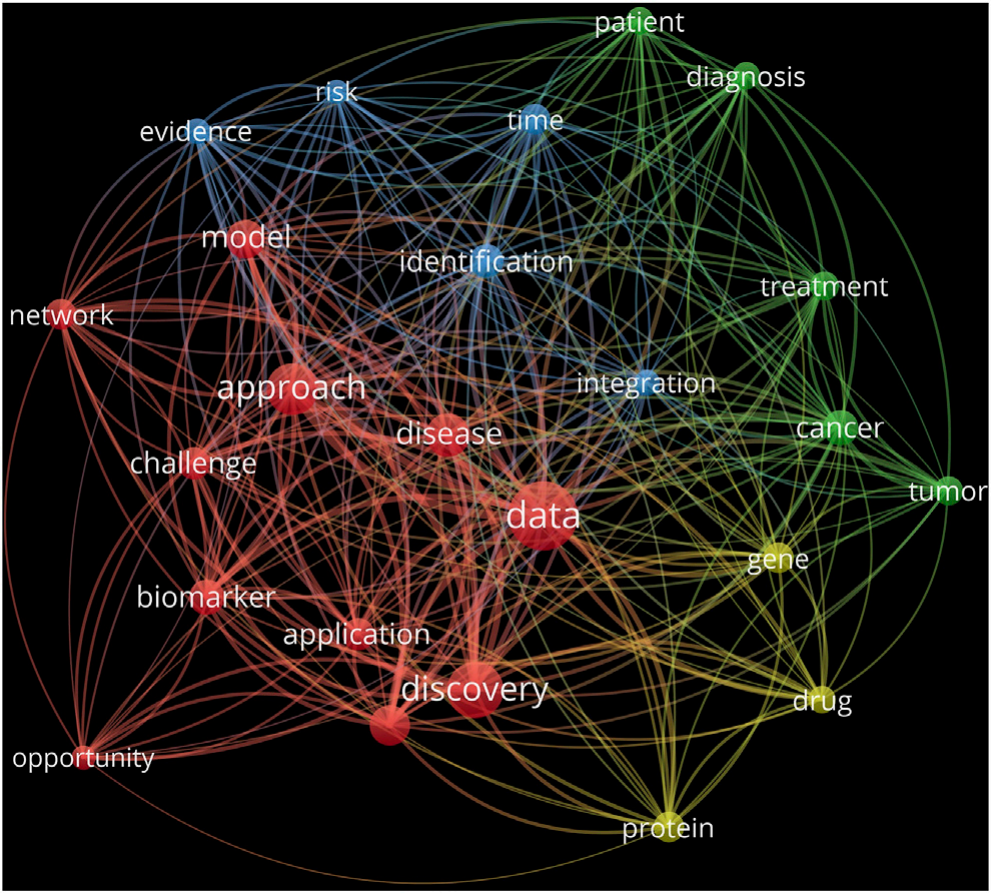
Bibliometric analysis of published studies related to the research topic theme. Network visualisation was generated by the use of VOSviewer 1.6.18.

This research topic collection focused on the theme of “bioinformatics and the translation of data-driven discoveries.” A PubMed (Fiorini et al., 2017) search with the theme as a keyword returned 56 published articles (as of 23 March 2022). A bibliometric network analysis herein of the articles’ title and abstract data using the VOSviewer tool (Perianes-Rodriguez et al., 2016) highlighted four overlapping clusters of top recurring terms (Figure 1). Each circle represents a term, while the size of a circle indicates the number of publications that have the corresponding term in their title or abstract. Terms that co-occur extensively tend to be located close to each other in the visualization. The red cluster was the largest and consisted of approach-related terms, such as network and model, with data, unsurprisingly, the most common term. The blue cluster appeared as an extension of the red cluster and emphasised various facets of the approach, such as identification and integration. The smallest, yellow cluster indicated the target focus of the approach, such as genes, proteins, and drugs. The green cluster, the same size as the blue, highlighted treatment-related terms, pivoting towards patient and cancer/tumour.

This Frontiers research topic was created in conjunction with the 19th International Conference on Bioinformatics (InCoB) 2020 (https://incob.apbionet.org/incob20) and was aligned with the theme of the conference. InCoB 2020 was held virtually from 25–29 November 2020 across Asia-Pacific and beyond. The conference included presentations of original research results, discussions in plenary sessions, poster sessions, workshops, software demos, and panel discussions related to the field of bioinformatics (APBioNET, 2021). The InCoB conference series is an annual, flagship conference of the Asia Pacific Bioinformatics Network (APBioNET; https://www.apbionet.org), an organisation that was established in 1999 with the simple mission of promoting bioinformatics in the region (Khan et al., 2013). The research topic collection received an encouraging tally of 20 submissions, within and outside the period of the conference. Covering the various facets of the theme, below we summarise the four submissions published as part of this collection.

Despite the progress in the reduction of the burden of Tuberculosis (TB) over the years, it remains a global health problem (WHO, 2021). This is compounded by the increase in the incidence of antibiotic-resistant (multidrug-resistant (MDR) and extensively drug-resistant (XDR)) forms of TB. The application of bioinformatics approaches to next-generation sequencing data of the disease agent, *Mycobacterium tuberculosis* (Mtb) can provide a high-throughput approach to better understand the resistance. Daniyarov et al. provided insights into genes associated with multi-drug resistance of Mtb through whole-genome sequencing, genotyping and characterisation of clinical isolates from patients in Kazakhstan. They identified several novel variants in drug-resistance genes. Correlation of the mutations to the phenotypic drug susceptibility profiles of the strains indicated a few with the potential to act as genetic determinants of resistance. The results merit further investigation, with the potential application to the design of intervention strategies.

While it is well established that genes are transcribed into mRNAs, which then get translated into proteins, it appears that these events can lead to “noise.” Chowdhury et al. studied this noise in bacterial gene expression, using combinatorial regulatory logic and have reported that *cis*-regulatory elements are crucial determinants of noise, which result in bacterial phenotypic variations. The results presented will enable the development of experimental strategies to dynamically follow gene transcription under different combinatorial regulatory mechanisms, to engineer novel microbial phenotypes.

Zeng et al. have successfully unravelled how the human transcription machinery can interpret the transcription start sites (TSSs) as either promoter or enhancer signals, using a deep learning (DL) method. The method uses a convolutional neural network (CNN) together with the saliency algorithm, which can capture high-order sequence features and outperform other classifiers. Furthermore, their detailed analysis of genomic features of the data arising from the FANTOM consortium has uncovered sequence differences downstream to the TSSs, where there is GC enrichment in the case of promoters, compared to enhancers. Their work has implications for understanding the foundations of RNA stability, from the sequence composition of flanking regions.

Seisinova et al. have identified potential prognostic and predisposition biomarkers of oesophageal carcinogenesis in predicting the early development of a tumour. They employed Independent Component Analysis (ICA), a matrix factorization method for reducing the data dimensions and performed a comprehensive transcriptomic analysis utilising the gene expression omnibus (GEO) datasets. Components or “*pseudocliques*” were mapped to the interacting partners of the proteins for constructing networks. The work forms the basis for a meta-analysis of oesophageal cancer transcriptomes, which warrants the need for wet-lab validation and further improvement in identifying candidate biomarkers.

In conclusion, the translation of biomedical data-driven discoveries is key and remains an important topic for future InCoB conferences. InCoB 2022 is planned to be held in Saudi Arabia, a first in the history of the conference, and will be hosted by King Abdullah University of Science and Technology (KAUST). The future holds promise with the integration of omics approaches, translating discoveries from bench to bedside.
